# Systemic immune-inflammation index as a potential biomarker of cardiovascular diseases: A systematic review and meta-analysis

**DOI:** 10.3389/fcvm.2022.933913

**Published:** 2022-08-08

**Authors:** Zhen Ye, Tingyi Hu, Jin Wang, Ruoyi Xiao, Xibei Liao, Mengsi Liu, Zhen Sun

**Affiliations:** ^1^Hengyang Medical School, University of South China, Hengyang, China; ^2^Department of Emergency Medicine, Xinqiao Hospital, Army Medical University, Chongqing, China

**Keywords:** cardiovascular disease (CVD), stroke, coronary heart disease, systemic immune-inflammation index (SII), inflammation, risk factor, biomarker, meta-analysis

## Abstract

**Background:**

Several studies have investigated the value of the systemic immune-inflammation index (SII) for predicting cardiovascular disease (CVD), but the results were inconsistent. Therefore, a meta-analysis and systematic review were conducted to assess the correlation between SII and risk of CVD.

**Materials and methods:**

Two investigators systematically searched PubMed, Embase, Web of Science, Cochrane library, and CINAHL databases to identify all studies that examined the association between SII levels and CVD. The risk estimates of CVD for people with high SII compared to those with low SII levels and the weighted mean difference (WMD) between the CVD and control groups were pooled using fixed- or random-effects models based on the heterogeneity test. We used the Newcastle-Ottawa Scale to assess the risk of bias in eligible studies, and the Grading of Recommendations Assessment, Development, and Evaluation (GRADE) system was applied to rate the certainty of evidence.

**Results:**

A total of 13 studies with 152,996 participants were included for analysis. The overall pooled results showed that higher SII was significantly associated with an increased risk of CVD (HR = 1.39, 95%CI: 1.20–1.61, *P* < 0.001). This increased risk could be observed in almost all CVD subtypes, including ischemic stroke (HR = 1.31, 95%CI: 1.06–1.63, *P* = 0.013), hemorrhagic stroke (HR = 1.22, 95%CI: 1.10–1.37, *P* < 0.001), myocardial infarction (HR = 1.11, 95%CI: 1.01–1.23, *P* = 0.027), and peripheral arterial disease (HR = 1.51, 95%CI: 1.18–1.93, *P* = 0.001). There were no significant but still similar trends in venous thrombosis (HR = 4.65, 95%CI: 0.66–32.71, *P* = 0.122), cerebral small vessel disease (HR = 1.09, 95%CI: 0.95–1.25, *P* = 0.233), and acute coronary syndrome (HR = 1.08, 95%CI: 0.96–1.22, *P* = 0.200). Furthermore, the pooled results showed that SII levels at the onset of CVD were significantly higher than that in the general population (WMD = 355.2, 95%CI: 234.8–475.6, *P* < 0.001), which was consistent across different CVD subtypes. The GRADE assessment suggested that the quality of current evidence from observational studies was low or very low.

**Conclusion:**

This study indicated that SII may be a potential biomarker for CVD development and elevated SII is associated with an increased risk of CVD. However, the quality of evidence is generally low. Additional well-designed studies are necessary to determine the optimal cutoff value and to characterize the benefited population.

## Graphical Abstract

The correlation between systemic immune-inflammation index (SII) and cardiovascular diseases (CVD).

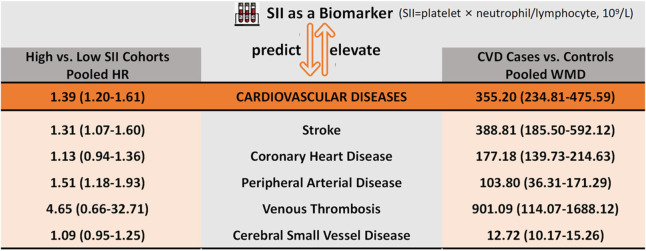

## Highlights

- Individuals with higher systemic immune-inflammation index (SII) had an increased risk of cardiovascular disease (CVD).
- Systemic immune-inflammation index at the onset of CVD was significantly higher than that in the general population.
- The level of evidence from observational studies is generally low.
- The optimal cutoff value for SII and characteristics of the benefited population remain undetermined.

## Introduction

Cardiovascular disease (CVD) is the leading cause of non-communicable diseases (NCDs) and is the largest single cause of death worldwide (1, 2). Approximately 18 million people die from CVD each year, accounting for half of all NCD deaths (3). According to statistics, the annual cost of CVD in the United States is US$378 billion (4). As a result, the United Nations has set a number of Sustainable Development Goals, including reducing premature deaths from NCDs by one-third by 2030 (5). To reduce the burden and damage caused by CVD, it is important to explore the correlation between various indicators and CVD risk to help identify people at high risk of CVD who need early intervention.

Stroke and ischemic heart disease are two major diseases that contribute to the burden of CVD and cause of disability-adjusted life years (6), and their major cause is atherosclerosis (7). Atherosclerosis is a systemic chronic inflammatory vascular disease, and thrombosis, oxidative stress, and endothelial damage may be the potential pathogenic mechanisms (8, 9). Studies have shown a profound link between immune and inflammatory responses in the development of atherosclerosis (8–10). The white blood cells that make up the immune system, including lymphocytes, neutrophils, monocytes, and macrophages, play different roles in atherosclerosis. For example, neutrophils can accelerate all stages of atherosclerosis by activating macrophages, promoting monocyte recruitment, and cytotoxicity, while lymphocytes regulate the inflammatory response and therefore have an anti-atherosclerotic effect (11, 12). There is also evidence that platelets adhere to the vessel wall to enhance leukocyte aggregation and initiate atherosclerotic progression before leukocytes invade the atherosclerotic plaque (13, 14). In recent years, a growing number of studies have explored some low-cost, validated, reproducible indicators of inflammation and immunity, such as the neutrophil-lymphocyte ratio (NLR) and platelet-lymphocyte ratio (PLR), with results suggesting that these combined markers are better predictors of CVD prognosis than single-cell counts (15–19). A new index, the systemic immune-inflammation index (SII), calculated as (neutrophil × platelet)/lymphocyte has recently been introduced for CVD, which can provide a relatively comprehensive reflection of the balance between host’s inflammation and immunity status (20); SII can be easily obtained by the routine blood test for complete blood count, the most commonly performed test in clinical practice. This marker was first proposed by Hu et al. (20) and has shown a higher predictive value for prognosis in the cancer field than other inflammatory factors, such as NLR and PLR (21, 22). Interestingly, recent studies have suggested that SII may be associated with prognosis and mortality in CVD (23).

Considering the importance of CVD risk identification and early intervention, some studies have explored the association between SII and risk of CVD, but no consistent conclusions have been reached. Therefore, we conducted a meta-analysis and systematic review to comprehensively examine the value of SII as an inexpensive and accessible indicator for predicting the risk of different CVD.

## Materials and methods

This study was reported based on the Preferred Reporting Items for Systematic Reviews and Meta-Analyses guidelines (24). The protocol of the study is not registered.

### Literature search

Two investigators (ZY and RX) searched the PubMed, Embase, Web of Science, Cochrane library, and CINAHL databases for relevant studies published from database inception to 30 September 2021, using search strategies formed by Medical Subject Headings combined with synonyms; the search was updated on 10 June 2022. In addition to the items for “cardiovascular disease,” some common specific CVD types, such as coronary artery disease, cerebrovascular accident, heart infarction, vein thrombosis, and peripheral vascular disease, were also added to the search formula. The detailed search strategy for each data item is presented in Supplementary Table 1.

### Eligibility criteria

According to the PICOS criteria defining the research questions, studies would be included if: (a) the study design was comparative, either the cohort or case-control study; (b) the study subjects are humans, with no restrictions on demographic characteristics, such as age, gender, or ethnicity; (c) for cohort studies, the population is divided into low-SII and high-SII level cohorts, and case-control studies should consist of CVD and non-CVD groups; (d) the correlation between different SII levels and CVD risk was assessed; risk ratios (RRs), odd ratios (ORs), or hazard ratios (HRs) were reported as effect sizes for this correlation, or sufficient data were provided to calculate these effect sizes. In addition, case-control studies that compared SII values between the non-CVD controls and CVD cases at the onset of cardiovascular events were also considered.

Commentaries, editorials, reviews, cellular and animal model experiments, reviews, expert opinion, meta-analyses, conference abstracts, or studies with duplicate and clearly problematic data were excluded.

### Study selection and quality assessment

Two authors (ZY and JW) screened the identified studies according to the inclusion and exclusion criteria. The retrieved records were preliminary screened by browsing titles and abstracts, and potentially eligible studies were read in full. Reasons for study exclusion were recorded and cross-checked. Any inconsistencies were resolved by consensus of all authors.

The quality of eligible studies was assessed by the Newcastle-Ottawa Scale (NOS) in terms of selection, comparability, and outcome/exposure. A study with an NOS score of <7 was considered to be a low-quality study; otherwise, it was considered to be at low risk of bias. We used the Grading of Recommendations Assessment, Development, and Evaluation (GRADE) system to evaluate the certainty of the evidence, mainly regarding study design, risk of bias, directness of the evidence, consistency and precision of effect estimates, publication bias, large effect, plausible confounding, and dose-response gradient (25, 26). Evidence from observational studies was considered low quality and was further reduced to very low if there were serious or very serious issues related to the risk of bias, inconsistency, indirectness, imprecision, and publication bias; if none of these aspects were reduced, three factors, namely, large effect, plausible confounding, and dose-response gradient, were considered for the possibility of upgrading the strength of the recommendations.

### Data extract

For eligible studies, one investigator (XL) extracted the data and another investigator (ZY) checked the accuracy. The following information was recorded: first author, publication year, study area, sample size, age, study design, population source, study period, cardiovascular events, SII cutoff, method of cutoff determination, adjusted/matched confounding factors, follow-up time, and effect size.

### Statistical analysis

The pooled HRs and corresponding 95%CI were calculated as the measure of the correlation between SII and CVD risk. In addition, we calculated the weighted mean difference (WMD) of SII values between the CVD and control groups to further validate the correlation. When the median, maximum, minimum, and/or quartiles of SII were provided in the original study, we used the method proposed by Wan et al. (27) to estimate the mean and standard deviation. Cochran’s *Q*-test and Higgins’ *I*^2^ statistics were used to assess heterogeneity between published studies. Heterogeneity was considered significant when *P* < 0.05 or *I*^2^ > 50%, in which case the random-effects model was used; otherwise, results from the fixed-effects model were reported. The sensitivity of the analyses was checked by excluding one study at a time and then combining the remaining studies; we also performed a stratified analysis based on region and sample size. Egger’s and Begg’s tests were used to quantitatively assess the risk of publication bias. Data analysis was completed using Stata MP/16.0 (StataCorp LLC, TX, United States); all *P*-values were two-tailed and *P* < 0.05 was considered statistically significant.

## Results

### Study characteristics

**FIGURE 1:**
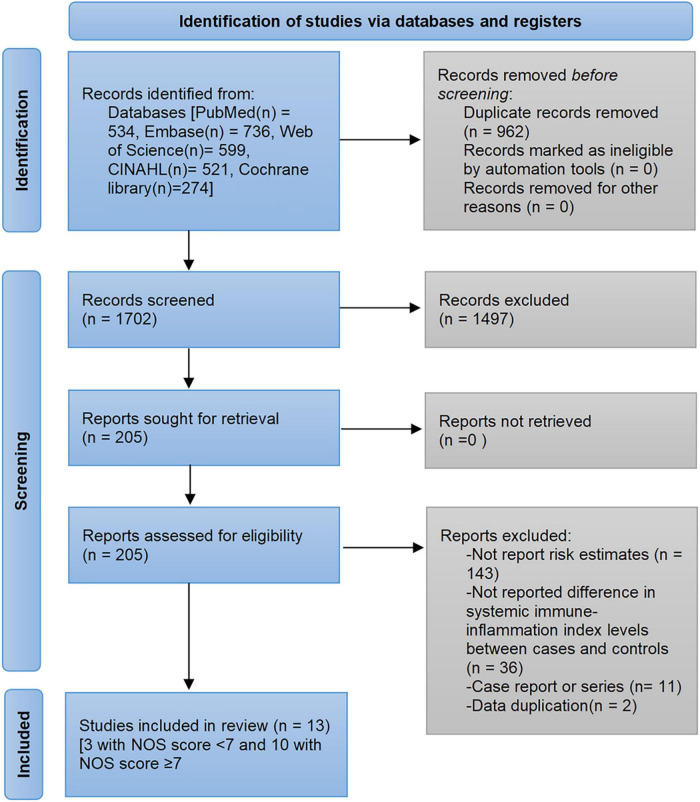
Flow diagram of the study selection process.

The pre-developed search strategy identified 2,664 records from PubMed, Embase, Web of Science, Cochrane library, and CINAHL databases. Based on the inclusion and exclusion criteria, a total of 13 studies were eligible for analysis (28–40). The flow diagram of the study selection process and exclusion reasons after full-reading are depicted in Figure 1. Of the 152,996 participants, 102,822 were male and the mean/median age ranged from 33 to 70 years. The published studies were conducted in four regions: China (28, 29, 31, 32, 34, 35, 39, 40), Turkey (30, 33, 38), Poland (36), and United States (37). The determination of SII cutoff values was based on ROC analysis, Youden index, and quartiles. Detailed characteristics of the included studies are shown in Table 1.

**TABLE 1:** Characteristics of studies investigating the relationship between systemic immune-inflammation index (SII) and cardiovascular disease (CVD).

References	Region	No. (M/F)	Age-year	High SII cohort/CVD group	Low SII cohort/control group	Design	Population	Cardiovas cular events	SII cut-off value	Identifi cation of cut-off value	Adjusted/matched confounding factor	Followed up-year
Zhang et al. (28)	China	150 (94/56)	Mean 38.37	90	60	Case-control study	General	CVT	496.07	Youden index.	White cells, neutrophils, lymphocytes, monocytes, monocyte/high-density lipoprotein ratio, and mean platelet volume	NP
Xu et al. (29)	China	13,929 (6,142/7,787)	Mean 62.56	10,446	3,483	Cohort study	Dongfeng-Tongji cohort	Stroke, CHD	223.08	Quartiles	Age, sex, test center, educational level, smoking status, drinking status, physical activity, BMI, drug use for thrombus treatment, family history of CHD or stroke, and hypertension, diabetes mellitus, and hyperlipidemia.	Median of 8.28 (max 8.98)
Tosu et al. (30)	Turkey	280 (119/161)	Mean 56.9	139	141	Case-control study	General	Isolated CAE	NA	NA	Age, gender, and body mass index	NP
Jin et al. (31)	China	85,154 (67,399/17,755)	Mean 48.6	60,344	20,054	Cohort study	Kailuan cohort	Stroke, MI	137.56	Quartiles	Age, gender, BMI, smoking, drinking, education, marriage, income level, physical activity, family history of cardiovascular disease, triglycerides, high-density lipoproteins, type 2 diabetes, hypertension, and C-reactive protein	10
Liu et al. (32)	China	395 (228/167)	Mean 62	285	110	Case-control study	General	CHD	439.44	ROC analysis	Age, presence of hypertension, presence of diabetes, smoking status, total white blood cell count, lymphocyte count, platelet count, high-density lipoprotein cholesterol level, creatinine level, cystatin C level, and C-reactive protein level	NP
Karahan et al. (33)	Turkey	102 (22/80)	Mean 33.25	51	51	Case-control study	Kara deniz Technical University Medical Faculty, Turkey,	CVST	NA	NA	NP	NP
Weng et al. (34)	China	1,091 (746/345)	Median 68.5/39	216	875	Case-control study	The Third Affiliated Hospital of Wenzhou Medical University	AIS	NA	NA	Age, sex, hypertension, diabetes, and hyperlipidemia	NP
Liu et al. (35)	China	1,179 (742/437)	Mean 45.6	362	817	Cohort study	SSIOS	DVT	1066	ROC curve	NP	NP
Morga et al. (36)	Poland	39 (14/25)	Median > 54	19	20	Case-control study	Caucasian	SAH	NA	NA	NP	NP
Zhang et al. (37)	United States	6,576 (3,389/3,187)	Mean 59.5	459	6,117	Cross-sectional study	NHANES	PAD	809.86	ROC curve	Age, sex, race, body mass index, diabetes mellitus, hypertension, and CHD	NA
Aydin et al. (38)	Turkey	379 (172/207)	Mean 52.5	49	330	Case-control study	Hypertensive patients	Stroke	NA	NA	NP	NP
Jiang et al. (39)	China	3,052 (1,419/1,633)	Mean 61.2	931	2,121	Cross-sectional study	Community-based population	CSVD	255.0	Quartiles	Age, sex, BMI, diabetes, hypertension, total cholesterol, high-density lipoprotein, low-density lipoprotein, fasting blood glucose, homocysteine, previous dyslipidemia, previous heart disease, current smoking, current drinking, previous antiplatelet, anticoagulant, antihypertensive, antidiabetic, and lipid-lowering drug	NA
Zhang et al. (40)	China	40,670 (22,336/18,334)	Median 70	11,610	29,060	Cohort study	Chinese PLA General Hospital*	Perioperative ischemic stroke	583.0	ROC analysis	Age, sex, BMI, ASA classification, hypertension, diabetes, prior ischemic stroke, coronary heart disease, arterial fibrillation, peripheral vascular disease, renal dysfunction, β-blockers medication, aspirin, preoperative hemoglobin, albumin, total bilirubin, prothrombin time, MAP, NLR, and PLR, surgical procedures, duration of procedures, estimated blood loss, MAP, crystalloid infusion, colloid infusion, blood transfusion, NSAIDs, glucocorticoid, opioid dose, volatile anesthetic	30 days

SII, systemic immune-inflammation index; CVD, cardiovascular disease; F/M, female/male; CVT, cerebral venous thrombosis; CAE, coronary artery ectasia; MI, myocardial infarction; CHD, coronary heart disease; CVST, cerebral venous sinus thrombosis; AIS, acute ischemic stroke; DVT, deep venous thrombosis; SAH, subarachnoid hemorrhage; PAD, peripheral arterial disease; CSVD, cerebral small vessel disease; ROC, receiver-operating characteristic; NHANES, National Health and Nutrition Examination Survey; SSIOS, database of surgical site infection in orthopedic surgery; ASA, American Society of Anesthesiologists; MAP, mean arterial pressure; NSAIDs, non-steroid anti-inflammatory drugs; NLR, neutrophil-lymphocyte ratio; PLR, platelet-to-lymphocyte ratio; NP, not reported; NA, not available.

*Patients who underwent non-cardiac surgery.

Ten studies had NOS scores between 7 and 9, indicating low risk of bias based on NOS scale. Three studies had NOS scores of 6, and the main risk of bias was a lack of comparability due to not adjusting for confounding factors (Supplementary Table 2).

### Association between systemic immune-inflammation index and cardiovascular diseases

**FIGURE 2:**
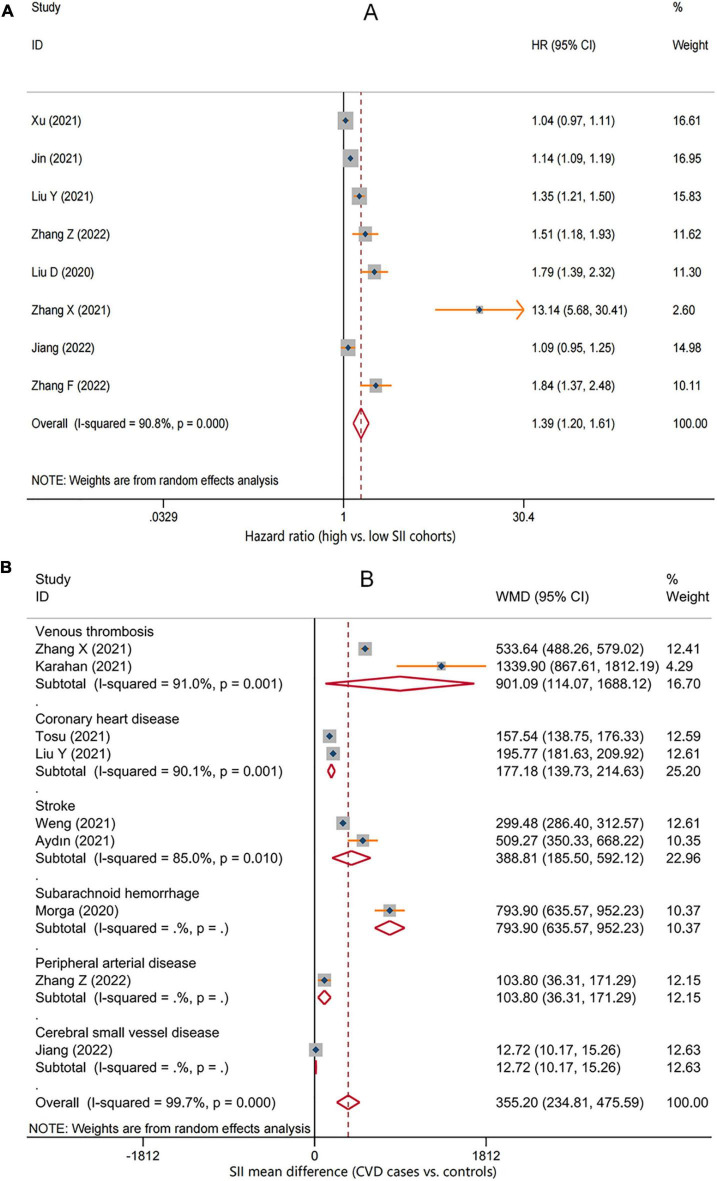
Forest plot for the associations between systemic immune-inflammation index (SII) and cardiovascular disease (CVD). **(A)** The relative risk of total CVD in high SII populations compared with low SII populations. **(B)** The weighted mean difference of SII levels in the patients with different CVD compared with the controls. HR, hazard ratio; WMD, weighted mean difference.

A total of eight studies reporting future CVD risk in cohorts with different SII levels were included in the analysis of the correlation between SII and CVD risk (28, 29, 31, 32, 35, 37, 39, 40). There was significant heterogeneity between studies (*I^2^* = 90.8%, *P* < 0.001), so a random-effects model was applied; the overall pooled result showed that higher SII was significantly associated with an increased risk of CVD (HR = 1.39, 95%CI: 1.20–1.61, *P* < 0.001) (Figure 2A). Subgroup analysis based on CVD types showed that the risk of all cardiovascular events tended to be higher in the high SII population. Except for cerebral small vessel disease (HR = 1.09, 95%CI: 0.95–1.25, *P* = 0.233), coronary heart disease (HR = 1.13, 95%CI: 0.94–1.36, *P* = 0.188), and venous thrombosis (HR = 4.65, 95%CI: 0.66–32.71, *P* = 0.122), there was a consistent statistical significance for the risk of ischemic stroke (HR = 1.31, 95%CI: 1.06–1.63, *P* = 0.013), hemorrhagic stroke (HR = 1.22, 95%CI: 1.10–1.37, *P* < 0.001), myocardial infarction (HR = 1.11, 95%CI: 1.01–1.23, *P* = 0.027), and peripheral arterial disease (HR = 1.51, 95%CI: 1.18–1.93, *P* = 0.001) (Table 2). Furthermore, nine studies reported differences in SII levels at the onset of CVD compared with the general population (28, 30, 32–34, 36–39), and all studies consistently suggested that SII levels at the onset of CVD were significantly higher than that in controls, with a median elevation range of 12–1,340. The random-effects model was used due to substantial heterogeneity (*I^2^* = 99.7%, *P* < 0.001), and the pooled WMD was 355.2 (95% CI: 234.8–475.6, *P* < 0.001) (Figure 2B), further confirming the correlation between SII and CVD development.

**TABLE 2:** The association between systemic immune-inflammation index (SII) and risk of specific cardiovascular disease (CVD) subtypes.

Subgroup	No. of studies	Hazard ratio	95% CI	*P*_*overalleffect*_	*I*^2^ static, *P*_*heterogeneity*_	Effects model
**Total stroke**	3	1.31	1.07–1.60	**0.010**	77.0%, 0.013	Random
Ischemic stroke	3	1.31	1.06–1.63	**0.013**	77.8%, 0.011	Random
Hemorrhagic stroke	2	1.22	1.10–1.37	**<0.001**	0.0%, 0.840	Fixed
**Total CHD**	3	1.13	0.94–1.36	0.188	91.4%, <0.001	Random
ACS	1	1.08	0.96–1.22	0.200	NA	NA
MI	1	1.11	1.01–1.23	**0.027**	NA	NA
**PAD**	1	1.51	1.18–1.93	**0.001**	NA	NA
**Venous thrombosis**	2	4.65	0.66–32.71	0.122	94.9%, <0.001	Random
**CSVD**	1	1.09	0.95–1.25	0.233	NA	NA

SII, systemic immune-inflammation index; CVD, cardiovascular disease; CHD, coronary heart disease; ACS, acute coronary syndrome; MI, myocardial infarction; PAD, peripheral arterial disease; CSVD, cerebral small vessel disease. Pvalues in bold indicate statistical significance.

### Sensitivity analysis and publication bias assessment

**FIGURE 3:**
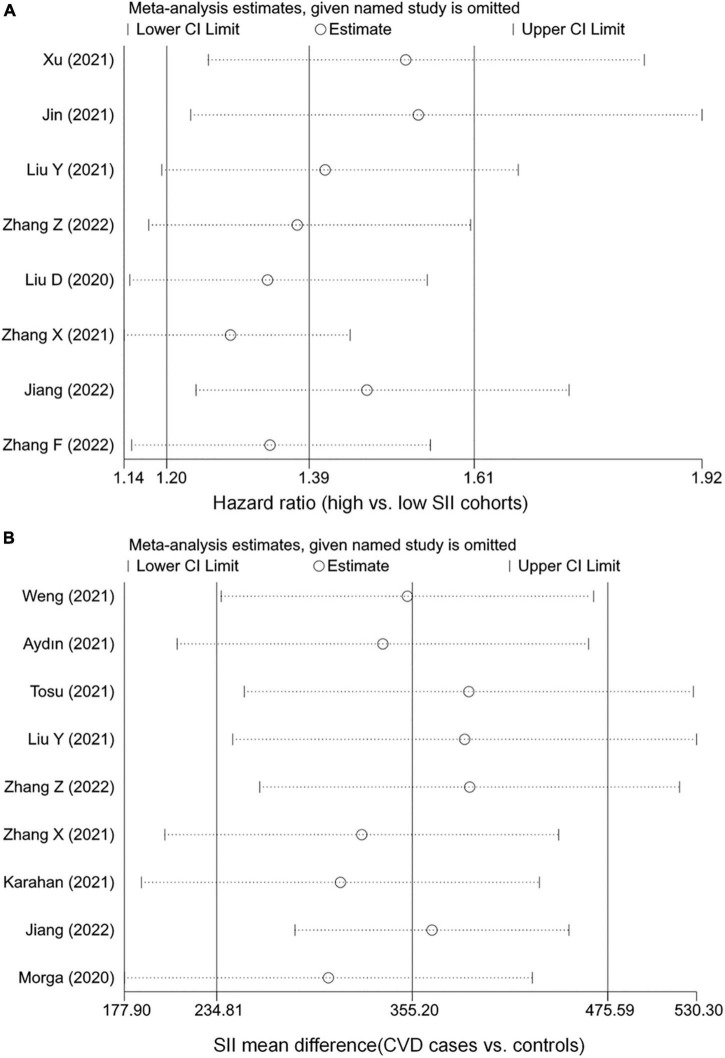
Sensitivity analysis of the associations between systemic immune-inflammation index (SII) and cardiovascular disease (CVD). **(A)** The hazard ratio of CVD. **(B)** The weighted mean difference of SII levels.

The robustness of the combined results was assessed by excluding one study at a time to observe its impact on the results. As shown in Figure 3, the conclusions remained unchanged after any individual studies were excluded, indicating that the current results are relatively stable. In addition, we conducted subgroup and meta-regression analyses based on study region and sample size. Although findings were unchanged across studies with different regions and sample sizes, representation of populations outside of China was relatively insufficient, and small sample size studies seemed to tend to report larger effect sizes. The meta-regressions suggested that region and sample size were not sources of heterogeneity, but this may be due to the lack of statistical power caused by the small number of studies (Supplementary Table 3).

The *P*-values for Begg’s and Egger’s tests were 1.000 and 0.781, respectively, indicating a low risk of publication bias and small study effects.

### GRADE assessments

The GRADE evidence profile table was generated with Guideline Development Tool (GRADEpro). Due to concerns over the vulnerability of observational studies to selection bias, the certainty of the evidence was initially rated as low quality for all endpoints, with coronary heart disease, stroke, and venous thrombosis downgraded one level to very low because of the potential risk of bias and/or inconsistency (Table 3).

**TABLE 3:** Quality of evidence of the association between systemic immune-inflammation index (SII) and cardiovascular disease (CVD).

Certainty assessment							Effect		Certainty	Importance
No. of studies	No. of participants	Risk of bias	Inconsistency	Indirectness	Imprecision	Other considerations*	Relative (95% CI)	Absolute (95% CI)		
Coronary heart disease										
3 Observational studies	99,478	Serious*^a^*	Very serious*^b^*	Not serious	Not serious	None	HR 1.13 (0.94 to 1.36)	1 fewer per 1,000 (from 1 fewer to 1 fewer)	⊕○○○ Very low	CRITICAL
Stroke										
3 Observational studies	139,753	Serious*^a^*	Very serious*^b^*	Not serious	Not serious	None	HR 1.31 (1.07 to 1.60)	7 more per 1,000 (from 2 more to 14 more)	⊕○○○ Very low	CRITICAL
Peripheral arterial disease										
1 Observational studies	6,576	Not serious	Not serious	Not serious	Not serious	None	HR 1.51 (1.18 to 1.93)	2 fewer per 1,000 (from 2 fewer to 1 fewer)	⊕⊕○○ Low	CRITICAL
Venous thrombosis										
2 Observational studies	1,329	Serious*^a^*	Very serious*^b^*	Not serious	Not serious	None	HR 4.65 (0.66 to 32.71)	5 fewer per 1,000 (from 33 fewer to 1 fewer)	⊕○○○ Very low	CRITICAL
Cerebral small vessel disease										
1 Observational studies	3,052	Not serious	Not serious	Not serious	Not serious	None	HR 1.09 (0.95 to 1.25)	1 fewer per 1,000 (from 1 fewer to 1 fewer)	⊕⊕○○ Low	CRITICAL

CI, confidence interval; HR, hazard ratio.

^a^Studies with some risks of bias were included in this analysis (NOS < 7).

^b^Considerable heterogeneity (*I*^2^ > 75%).

*Publication bias, large effect, plausible confounding, and dose response gradient.

High quality: Further research is very unlikely to change our confidence in the estimate of effect.

Moderate quality: Further research is likely to have an important impact on our confidence in the estimate of effect and may change the estimate.

Low quality: Further research is very likely to have an important impact on our confidence in the estimate of effect and is likely to change the estimate.

Very low quality: Any estimate of effect is very uncertain.

## Discussion

To the best of our knowledge, this is the first study to focus on examining the association between SII and CVD risk. SII is a new inflammatory index based on platelet, neutrophil, and lymphocyte counts that reflects the host’s inflammatory and immune status. We found a significantly higher risk of CVD in people with high SII levels; this risk can be observed in almost every type of CVD. SII values were also significantly increased at the onset of various CVD. These findings suggest that SII may serve as an indicator to identify people at high risk of CVD and contribute to early diagnosis. However, due to the nature of observational studies, the quality of the current evidence is rated as low or very low.

Cardiovascular disease encompasses a wide range of diseases with diverse and non-specific clinical presentations, making it easy to delay diagnosis and misdiagnosis, which leads to deterioration of the patient’s condition. Therefore, exploring a simple and widely available index to predict CVD is crucial. A large body of evidence suggests that chronic inflammation plays a key role in the pathogenesis of CVD (41–43). Specifically, neutrophils secrete inflammatory mediators that can lead to endothelial dysfunction and vessel wall degeneration (44). Platelets may release some chemokines, proinflammatory cytokines, and platelet-derived growth factors that promote endothelial cell depletion (45, 46). In addition, neutrophils promote atherosclerosis and thrombosis through interactions with platelets, proteolytic cleavage of coagulation factors, release of prothrombotic molecules, and promotion of monocyte infiltration, ultimately leading to cardiovascular events (47). Conversely, lymphocytes can regulate inflammatory responses and have anti-atherosclerotic effects. However, due to the diversity of T cell types, their roles are also diverse (48). For example, CD8+, CD4+, γδ T cells exacerbate stroke injury by secreting interferon-γ and IL-17, while T regulatory cells (CD4+, CD25+, Foxp3+, and T regulatory cell) release the anti-inflammatory cytokine IL-10, which is beneficial to the condition (49–51). Therefore, the detection of lymphocyte subpopulations may further enhance the strength of the association.

The SII has unique advantages over other biological indicators, such as NLR, PLR, and C-reactive protein (CRP). Liu et al. (32) found that SII had better predictive power for the occurrence of coronary artery disease compared with NLR, PLR, and C-reactive protein (CRP). Xu et al. (29) observed a significant linear dose-response association between SII and risk of total stroke. SII appears to be more stable than blood counts alone, and these counts are susceptible to various factors, such as dehydration and fluid overload (52, 53). Of note, among these diseases, SII was correlated most significantly with cerebral venous thrombosis, and the study by Zhang et al. (28) found that SII could be used as a strong independent predictor in multivariable regression analysis (SII degree, OR = 13.136, 95%CI 5.675–30.407, *P* < 0.001). This means that in patients with unexplained headache and a normal plain CT, SII can help clinicians to quickly infer cerebral venous thrombosis and determine its severity and the necessity for immediate MRI/MRV confirmation, which can help to shorten the time between symptom onset and diagnosis and reduce misdiagnosis (28). Furthermore, Zhang et al. (40) found that preoperative SII can help predict the risk of perioperative ischemic stroke in non-cardiac surgery patients, suggesting that physicians can formulate more rational perioperative prevention and treatment plans based on SII levels. In the chronic disease population, Xu et al. (29) found a stronger positive association between SII and total CVD in patients with hypertension or diabetes. This may be related to endothelial dysfunction, inflammatory infiltration, and vascular remodeling caused by long-term inflammation in patients with diabetes or hypertension (54).

In addition to the correlation with CVD risk, SII has been found to predict the severity, complications, and mortality of CVD. For example, Zhang et al. (28) found that SII was positively correlated with the baseline National Institutes of Health Stroke Score (NIHSS); Weng et al. (34) demonstrated that SII was associated with stroke severity and that SII level was an independent prognostic indicator of poor prognosis at 3 months. Interestingly, Liu et al. (32) found by correlation analysis that SII was a better predictor of coronary artery disease severity than NLR, PLR, and CRP levels.

The SII reflects a significant association of the host’s inflammatory and immune status with the risk of CVD. People with elevated SII on physical examinations may be recommended to adopt a lifestyle that can reduce inflammation, such as smoking cessation, weight control, reasonable emotional regulation, reduced sedentary behavior, regular exercise and rest, and reduction of saturated fat and refined carbohydrate content in the diet (55–57). Moreover, reminding the general population with high SII levels to increase the frequency of medical screening as appropriate may help patients benefit from secondary prevention treatment (58).

The main limitation of this study is the small number of studies in some CVD subtypes resulting in conservative 95%CI. For example, for venous thrombosis, although a strong correlation was found, it was not significant, which may be due to insufficient statistical power. Second, populations outside of China are relatively underrepresented, and more studies from other geodemographic settings are necessary to examine possible regional specificities. Third, although Egger’s and Begg’s tests did not show significance, small study effects may still be present; studies with smaller sample sizes seemed to tend to report stronger correlations. Fourth, the limited data did not support a more detailed stratified analysis to identify populations that would benefit more from SII indicators. Furthermore, current studies on the association between SII and CVD risk used different SII cutoff values, which need to be standardized before SII can be widely used.

In conclusion, our study indicated that SII may be a potential predictor of CVD. Healthcare providers need to be concerned about CVD risk in people with high SII and appropriate interventions may be needed. Due to the nature of observational studies, the quality of the evidence was graded from low to very low, which means that further research on the subject is necessary to draw solid conclusions. In addition, future studies should further determine the optimal cutoff value, characterize the benefited population, and investigate whether SII can be used in conjunction with other known CVD risk factors to build an accurate CVD risk assessment system.

## Data availability statement

The original contributions presented in this study are included in the article/Supplementary material, further inquiries can be directed to the corresponding authors.

## Author contributions

ZS and ML: conception, design, and critical revision of the manuscript. ZY, XL, JW, and RX: database search, literature review, and data extraction. XL and TH: statistical analysis. ZY, TH, XL, JW, and RX: drafting of the manuscript. All authors contributed to the article and approved the submitted version.
