# Supplementary material for: Systemic immune-inflammation index as a potential biomarker of cardiovascular diseases: A systematic review and meta-analysis
Source: Front Cardiovasc Med. 2022 Aug 8;9:933913. doi: 10.3389/fcvm.2022.933913 (PMC9393310; doi:10.3389/fcvm.2022.933913)
Supplement: Supplementary file 1 [file Data_Sheet_1.doc]

| **TABLE S1**. The specific search strategy for each database | |
| --- | --- |
| **Web of science** | TS=((systemic immune inflammatory index OR systemic immune-inflammatory index OR SII OR Systemic-immune-inflammation index OR Systemic immune-inflammation index OR neutrophil ×platelets/lymphocyte) **AND** ((cardiovascular disease OR angiocardiopathy OR angiocardiovascular disease OR cardiovascular complication OR cardiovascular diseases OR cardiovascular disorder OR cardiovascular disturbance OR cardiovascular lesion or cardiovascular syndrome OR cardiovascular vegetative disorder or complication, cardiovascular OR disease, cardiovascular OR major adverse cardiovascular even ) OR (coronary arte ry disease or coronary disease or multivessel coronary artery disease) or (coronary artery disease OR coronary disease OR multivessel coronary artery disease) or (heart disease or cardiac anomaly or cardiac disease or cardiac disturbance or cardiopathy or heart deficiency or heart deformity or heart diseases or heart disorder or heart dysfunction) or (cerebrovascular accident or accident, cerebrovascular or acute cerebrovascular lesion or acute focal cerebral vasculopathy or acute stroke or apoplectic stroke or apoplexia or apoplexy or blood flow disturbance, brain or brain accident or brain attack or brain blood flow disturbance or brain insult or brain insultus or brain vascular accident or cerebral apoplexia or cerebral insult or cerebral stroke or cerebral vascular accident or cerebral vascular insufficiency or cerebro vascular accident or cerebrovascular arrest or cerebrovascular failure or cerebrovascular injury or cerebrovascular insufficiency or cerebrovascular insult or cerebrum vascular accident or cryptogenic stroke or CVA or ischaemic seizure or ischemic seizure or stroke or thrombotic stroke) or (cerebrovascular disease or brain angiopathy or brain circulation failure or brain vascular disease or brain vasculopathy or cerebral small vessel disease or cerebral small vessel diseases or cerebral vascular disease or cerebral vascular disorder or cerebral vascular disturbance or cerebral vascular lesion or cerebral vasculopathy or cerebrovascular damage or cerebrovascular disorder or cerebrovascular disorders or cerebrovascular lesion or cerebrovascular pathology or cerebrovascular syndrome) or (rheumatic heart disease or heart disease, rheumatic or rheumatic cardiac disease or rheumatic cardiopathy or rheumatic valve disease or rheumatic valvular disease or rheumatoid heart disease) or (atrial fibrillation or auricular fibrilation or auricular fibrillation or cardiac atrial fibrillation or cardiac atrium fibrillation or fibrillation, heart atrium or heart atrial fibrillation or heart atrium fibrillation or heart fibrillation atrium or non-valvular atrial fibrillation or nonvalvular atrial fibrillation) or (heart infarction or cardiac infarct or cardiac infarction or cardial infarct or heart attack or heart infarct or heart micro infarction or heart muscle infarction or infarction, heart or myocardial infarct or myocardial infarction or myocardium infarct or myocardium infarction or premonitory infarction sign or second heart attack or subendocardial infarction or transmural cardiac infarction or transmural heart infarction or transmural infarction, heart) or (heart muscle ischemia or acute heart muscle ischaemia or acute heart muscle ischemia or cardiac ischaemia or cardiac ischemia or cardiac muscle ischaemia or cardiac muscle ischemia or coronary artery ischaemia or coronary artery ischemia or coronary ischaemia or coronary ischemia or coronary syndrome or heart anoxia or heart hypoxia or heart ischaemia or heart ischaemic arrest or heart ischaemic attack or heart ischaemic time or heart ischemia or heart ischemic arrest or heart ischemic attack or heart ischemic time or heart muscle hypoxia or heart muscle ischaemia or heart muscle ischaemia, subepicardial or heart muscle ischemia, subepicardial or heart transient ischaemic attack or heart transient ischemic attack or hypoxia, heart or hypoxic heart or ischaemic heart or ischaemic heart arrest or ischaemic myocardium or ischemic heart or ischemic heart arrest or ischemic myocardium or myocardial anoxia or myocardial hypoxia or myocardial ischaemia or myocardial ischemia or myocardium hypoxia or myocardium ischaemia or myocardium ischemia or subendocardial ischaemia or subendocardial ischemia or transient ischaemic attack, heart or transient ischemic attack, heart) or (cardiovascular event or cardiovascular events) or (heart failure or backward failure, heart or cardiac backward failure or cardiac decompensation or cardiac failure or cardiac incompetence or cardiac insufficiency or cardiac stand still or cardial decompensation or cardial insufficiency or chronic heart failure or chronic heart insufficiency or decompensatio cordis or decompensation, heart or heart backward failure or heart decompensation or heart incompetence or heart insufficiency or insufficientia cardis or myocardial failure or myocardial insufficiency) or (cor pulmonale or cardiac disease, pulmonary or chronic cor pulmonale or corpulmonale or heart disease, pulmonary or pulmonary cardiac disease or pulmonary heart disease) or (lung embolism or chronic lung embolism or embolism, lung or lung embolization or lung embolus or lung embolus recurrence or lung emboly or lung microembolism or lung microembolization or lung microembolus or lung thromboembolism or microembolus, lung or pulmonary embolism or pulmonary embolization or pulmonary embolus or pulmonary microembolism or pulmonary thromboembolic disease or pulmonary thromboembolism or thromboembolism, lung) or (cardiovascular risk or risk, cardiovascular) or (vein thrombosis or phlebo-thrombosis or phlebothrombosis or thrombosis, venous or vena thrombosis or venothrombosis or venothrombotic event or venous thrombosis) or (macrovascular diseases) or (peripheral vascular disease or peripheral arteriopathy or peripheral blood vessel disease or peripheral vascular diseases or peripheral vascular disorder or peripheral vasculopathy or peripheral vessel disease) or (cardiocerebrovascular disease) or (cardiovascular death))) |
| **Embase** | ('systemic immune inflammatory index'/exp OR 'systemic immune inflammatory index':ab,ti OR ('systemic':ab,ti AND ('immune'/exp OR 'immune':ab,ti ) AND 'inflammatory':ab,ti AND ('index'/exp OR 'index':ab,ti ))) **AND** (('cardiovascular disease'/exp or 'angiocardiopathy':ab,ti or 'angiocardiovascular disease':ab,ti or 'cardiovascular complication':ab,ti or 'cardiovascular diseases':ab,ti or 'cardiovascular disorder':ab,ti or 'cardiovascular disturbance':ab,ti or 'cardiovascular lesion':ab,ti or 'cardiovascular syndrome':ab,ti or 'cardiovascular vegetative disorder':ab,ti or 'complication, cardiovascular':ab,ti or 'disease, cardiovascular':ab,ti or 'major adverse cardiovascular event':ab,ti ) or ('coronary arte ry disease'/exp or 'coronary disease':ab,ti or 'multivessel coronary artery disease':ab,ti ) or ('coronary artery disease'/exp or 'coronary disease':ab,ti or 'multivessel coronary artery disease':ab,ti ) or ('heart disease'/exp or 'cardiac anomaly':ab,ti or 'cardiac disease':ab,ti or 'cardiac disturbance':ab,ti or 'cardiopathy':ab,ti or 'heart deficiency':ab,ti or 'heart deformity':ab,ti or 'heart diseases':ab,ti or 'heart disorder':ab,ti or 'heart dysfunction':ab,ti ) or ('cerebrovascular accident'/exp or 'accident, cerebrovascular':ab,ti or 'acute cerebrovascular lesion':ab,ti or 'acute focal cerebral vasculopathy':ab,ti or 'acute stroke':ab,ti or 'apoplectic stroke':ab,ti or 'apoplexia':ab,ti or 'apoplexy':ab,ti or 'blood flow disturbance, brain':ab,ti or 'brain accident':ab,ti or 'brain attack':ab,ti or 'brain blood flow disturbance':ab,ti or 'brain insult':ab,ti or 'brain insultus':ab,ti or 'brain vascular accident':ab,ti or 'cerebral apoplexia':ab,ti or 'cerebral insult':ab,ti or 'cerebral stroke':ab,ti or 'cerebral vascular accident':ab,ti or 'cerebral vascular insufficiency':ab,ti or 'cerebro vascular accident':ab,ti or 'cerebrovascular arrest':ab,ti or 'cerebrovascular failure':ab,ti or 'cerebrovascular injury':ab,ti or 'cerebrovascular insufficiency':ab,ti or 'cerebrovascular insult':ab,ti or 'cerebrum vascular accident':ab,ti or 'cryptogenic stroke':ab,ti or 'CVA':ab,ti or 'ischaemic seizure':ab,ti or 'ischemic seizure':ab,ti or 'stroke':ab,ti or 'thrombotic stroke':ab,ti ) or ('cerebrovascular disease'/exp or 'brain angiopathy':ab,ti or 'brain circulation failure':ab,ti or 'brain vascular disease':ab,ti or 'brain vasculopathy':ab,ti or 'cerebral small vessel disease':ab,ti or 'cerebral small vessel diseases':ab,ti or 'cerebral vascular disease':ab,ti or 'cerebral vascular disorder':ab,ti or 'cerebral vascular disturbance':ab,ti or 'cerebral vascular lesion':ab,ti or 'cerebral vasculopathy':ab,ti or 'cerebrovascular damage':ab,ti or 'cerebrovascular disorder':ab,ti or 'cerebrovascular disorders':ab,ti or 'cerebrovascular lesion':ab,ti or 'cerebrovascular pathology':ab,ti or 'cerebrovascular syndrome':ab,ti ) or ('rheumatic heart disease'/exp or 'heart disease, rheumatic':ab,ti or 'rheumatic cardiac disease':ab,ti or 'rheumatic cardiopathy':ab,ti or 'rheumatic valve disease':ab,ti or 'rheumatic valvular disease':ab,ti or 'rheumatoid heart disease':ab,ti ) or ('atrial fibrillation'/exp or 'auricular fibrilation':ab,ti or 'auricular fibrillation':ab,ti or 'cardiac atrial fibrillation':ab,ti or 'cardiac atrium fibrillation':ab,ti or 'fibrillation, heart atrium':ab,ti or 'heart atrial fibrillation':ab,ti or 'heart atrium fibrillation':ab,ti or 'heart fibrillation atrium':ab,ti or 'non-valvular atrial fibrillation':ab,ti or 'nonvalvular atrial fibrillation':ab,ti ) or ('heart infarction'/exp or 'cardiac infarct':ab,ti or 'cardiac infarction':ab,ti or 'cardial infarct':ab,ti or 'heart attack':ab,ti or 'heart infarct':ab,ti or 'heart micro infarction':ab,ti or 'heart muscle infarction':ab,ti or 'infarction, heart':ab,ti or 'myocardial infarct':ab,ti or 'myocardial infarction':ab,ti or 'myocardium infarct':ab,ti or 'myocardium infarction':ab,ti or 'premonitory infarction sign':ab,ti or 'second heart attack':ab,ti or 'subendocardial infarction':ab,ti or 'transmural cardiac infarction':ab,ti or 'transmural heart infarction':ab,ti or 'transmural infarction, heart':ab,ti ) or ('heart muscle ischemia'/exp or 'acute heart muscle ischaemia':ab,ti or 'acute heart muscle ischemia':ab,ti or 'cardiac ischaemia':ab,ti or 'cardiac ischemia':ab,ti or 'cardiac muscle ischaemia':ab,ti or 'cardiac muscle ischemia':ab,ti or 'coronary artery ischaemia':ab,ti or 'coronary artery ischemia':ab,ti or 'coronary ischaemia':ab,ti or 'coronary ischemia':ab,ti or 'coronary syndrome':ab,ti or 'heart anoxia':ab,ti or 'heart hypoxia':ab,ti or 'heart ischaemia':ab,ti or 'heart ischaemic arrest':ab,ti or 'heart ischaemic attack':ab,ti or 'heart ischaemic time':ab,ti or 'heart ischemia':ab,ti or 'heart ischemic arrest':ab,ti or 'heart ischemic attack':ab,ti or 'heart ischemic time':ab,ti or 'heart muscle hypoxia':ab,ti or 'heart muscle ischaemia':ab,ti or 'heart muscle ischaemia, subepicardial':ab,ti or 'heart muscle ischemia, subepicardial':ab,ti or 'heart transient ischaemic attack':ab,ti or 'heart transient ischemic attack':ab,ti or 'hypoxia, heart':ab,ti or 'hypoxic heart':ab,ti or 'ischaemic heart':ab,ti or 'ischaemic heart arrest':ab,ti or 'ischaemic myocardium':ab,ti or 'Ischemic heart':ab,ti or 'ischemic heart arrest':ab,ti or 'ischemic myocardium':ab,ti or 'myocardial anoxia':ab,ti or 'myocardial hypoxia':ab,ti or 'myocardial ischaemia':ab,ti or 'myocardial ischemia':ab,ti or 'myocardium hypoxia':ab,ti or 'myocardium ischaemia':ab,ti or 'myocardium ischemia':ab,ti or 'subendocardial ischaemia':ab,ti or 'subendocardial ischemia':ab,ti or 'transient ischaemic attack, heart':ab,ti or 'transient ischemic attack, heart':ab,ti ) or ('cardiovascular event':ab,ti or 'cardiovascular events':ab,ti ) or ('heart failure'/exp or 'backward failure, heart':ab,ti or 'cardiac backward failure':ab,ti or 'cardiac decompensation':ab,ti or 'cardiac failure':ab,ti or 'cardiac incompetence':ab,ti or 'cardiac insufficiency':ab,ti or 'cardiac stand still':ab,ti or 'cardial decompensation':ab,ti or 'cardial insufficiency':ab,ti or 'chronic heart failure':ab,ti or 'chronic heart insufficiency':ab,ti or 'decompensatio cordis':ab,ti or 'decompensation, heart':ab,ti or 'heart backward failure':ab,ti or 'heart decompensation':ab,ti or 'heart incompetence':ab,ti or 'heart insufficiency':ab,ti or 'insufficientia cardis':ab,ti or 'myocardial failure':ab,ti or 'myocardial insufficiency':ab,ti ) or ('cor pulmonale'/exp or 'cardiac disease, pulmonary':ab,ti or 'chronic cor pulmonale':ab,ti or 'corpulmonale':ab,ti or 'heart disease, pulmonary':ab,ti or 'pulmonary cardiac disease':ab,ti or 'pulmonary heart disease':ab,ti ) or ('lung embolism'/exp or 'chronic lung embolism':ab,ti or 'embolism, lung':ab,ti or 'lung embolization':ab,ti or 'lung embolus':ab,ti or 'lung embolus recurrence':ab,ti or 'lung emboly':ab,ti or 'lung microembolism':ab,ti or 'lung microembolization':ab,ti or 'lung microembolus':ab,ti or 'lung thromboembolism':ab,ti or 'microembolus, lung':ab,ti or 'pulmonary embolism':ab,ti or 'pulmonary embolization':ab,ti or 'pulmonary embolus':ab,ti or 'pulmonary microembolism':ab,ti or 'pulmonary thromboembolic disease':ab,ti or 'pulmonary thromboembolism':ab,ti or 'thromboembolism, lung':ab,ti ) or ('cardiovascular risk'/exp or 'risk, cardiovascular':ab,ti ) or ('vein thrombosis'/exp or 'phlebo-thrombosis':ab,ti or 'phlebothrombosis':ab,ti or 'thrombosis, venous':ab,ti or 'vena thrombosis':ab,ti or 'venothrombosis':ab,ti or 'venothrombotic event':ab,ti or 'venous thrombosis':ab,ti ) or ('macrovascular diseases':ab,ti ) or ('peripheral vascular disease'/exp or 'peripheral arteriopathy':ab,ti or 'peripheral blood vessel disease':ab,ti or 'peripheral vascular diseases':ab,ti or 'peripheral vascular disorder':ab,ti or 'peripheral vasculopathy':ab,ti or 'peripheral vessel disease':ab,ti ) or ('cardiocerebrovascular disease':ab,ti ) or ('cardiovascular death':ab,ti )) |
| **PubMed** | (systemic immune inflammatory index or systemic immune-inflammatory index or SII or Systemic-immune-inflammation index OR Systemic immune-inflammation index OR neutrophil × platelets/ lymphocyte) **AND** (("Cardiovascular Diseases"[Mesh] OR Cardiovascular Disease or Disease, Cardiovascular OR Diseases, Cardiovascular) or ("Coronary Disease"[Mesh] or Coronary Diseases OR Disease, Coronary OR Diseases, Coronary or Coronary Heart Disease OR Coronary Heart Diseases OR Disease, Coronary Heart OR Diseases, Coronary Heart OR Heart Disease, Coronary OR Heart Diseases, Coronary OR coronary artery disease) OR ("Heart Diseases"[Mesh] OR Heart Disease OR Cardiac Diseases OR Cardiac Disease OR Cardiac Disorders OR Cardiac Disorder OR Heart Disorders OR Heart Disorder) OR ("Stroke"[Mesh] OR Strokes OR Cerebrovascular Accident OR Cerebrovascular Accidents OR CVA (Cerebrovascular Accident) OR CVAs (Cerebrovascular Accident) OR Cerebrovascular Apoplexy OR Apoplexy, Cerebrovascular OR Vascular Accident, Brain OR Brain Vascular Accident OR Brain Vascular Accidents OR Vascular Accidents, Brain OR Cerebrovascular Stroke or Cerebrovascular Strokes OR Stroke, Cerebrovascular OR Strokes, Cerebrovascular or Apoplexy or Cerebral Stroke OR Cerebral Strokes OR Stroke, Cerebral OR Strokes, Cerebral or Stroke, Acute or Acute Stroke OR Acute Strokes OR Strokes, Acute or Cerebrovascular Accident, Acute OR Acute Cerebrovascular Accident OR Acute Cerebrovascular Accidents OR Cerebrovascular Accidents, Acute) OR ("Cerebrovascular Disorders"[Mesh] OR Cerebrovascular Disorder OR Vascular Diseases, Intracranial OR Intracranial Vascular Disease OR Intracranial Vascular Diseases OR Vascular Disease, Intracranial OR Intracranial Vascular Disorders OR Intracranial Vascular Disorder OR Vascular Disorder, Intracranial OR Vascular Disorders, Intracranial OR Cerebrovascular Diseases OR Cerebrovascular Disease OR Disease, Cerebrovascular OR Diseases, Cerebrovascular OR Brain Vascular Disorders OR Brain Vascular Disorder OR Vascular Disorder, Brain OR Vascular Disorders, Brain OR Cerebrovascular Occlusion OR Cerebrovascular Occlusions OR Occlusion, Cerebrovascular OR Occlusions, Cerebrovascular OR Cerebrovascular Insufficiency OR Cerebrovascular Insufficiencies OR Insufficiencies, Cerebrovascular OR Insufficiency, Cerebrovascular) OR ("Rheumatic Heart Disease"[Mesh] OR Disease, Rheumatic Heart OR Diseases, Rheumatic Heart OR Heart Disease, Rheumatic OR Heart Diseases, Rheumatic OR Rheumatic Heart Diseases OR Bouillaud Disease OR Disease, Bouillaud OR Bouillaud's Disease OR Bouillauds Disease OR Disease, Bouillaud's) OR ("Atrial Fibrillation"[Mesh] OR Atrial Fibrillations OR Fibrillation, Atrial OR Fibrillations, Atrial OR Auricular Fibrillation OR Auricular Fibrillations OR Fibrillation, Auricular OR Fibrillations, Auricular OR Persistent Atrial Fibrillation OR Atrial Fibrillation, Persistent OR Atrial Fibrillations, Persistent OR Fibrillation, Persistent Atrial OR Fibrillations, Persistent Atrial OR Persistent Atrial Fibrillations OR Familial Atrial Fibrillation OR Atrial Fibrillation, Familial OR Atrial Fibrillations, Familial OR Familial Atrial Fibrillations OR Fibrillation, Familial Atrial OR Fibrillations, Familial Atrial OR Paroxysmal Atrial Fibrillation OR Atrial Fibrillation, Paroxysmal OR Atrial Fibrillations, Paroxysmal OR Fibrillation, Paroxysmal Atrial OR Fibrillations, Paroxysmal Atrial OR Paroxysmal Atrial Fibrillations) OR ("Myocardial Infarction"[Mesh] OR Infarction, Myocardial OR Infarctions, Myocardial OR Myocardial Infarctions OR Cardiovascular Stroke OR Cardiovascular Strokes OR Stroke, Cardiovascular OR Strokes, Cardiovascular OR Myocardial Infarct OR Infarct, Myocardial OR Infarcts, Myocardial OR Myocardial Infarcts OR Heart Attack OR Heart Attacks) OR ("Myocardial Ischemia"[Mesh] OR Ischemia, Myocardial OR Ischemias, Myocardial OR Myocardial Ischemias OR Ischemic Heart Disease OR Heart Disease, Ischemic OR Disease, Ischemic Heart OR Diseases, Ischemic Heart OR Heart Diseases, Ischemic OR Ischemic Heart Diseases) OR (cardiovascular event OR cardiovascular events) OR ("Heart Failure"[Mesh] OR Cardiac Failure OR Heart Decompensation OR Decompensation, Heart OR Heart Failure, Right-Sided OR Heart Failure, Right Sided OR Right-Sided Heart Failure OR Right Sided Heart Failure OR Myocardial Failure OR Congestive Heart Failure OR Heart Failure, Congestive OR Heart Failure, Left-Sided OR Heart Failure, Left Sided OR Left-Sided Heart Failure OR Left Sided Heart Failure) OR ("Pulmonary Heart Disease"[Mesh] OR Heart Disease, Pulmonary OR Heart Diseases, Pulmonary OR Disease, Pulmonary Heart OR Cor Pulmonale OR Diseases, Pulmonary Heart OR Pulmonary Heart Diseases) OR ("Pulmonary Embolism"[Mesh] OR Pulmonary Embolisms OR Embolism, Pulmonary OR Embolisms, Pulmonary OR Pulmonary Thromboembolisms OR Pulmonary Thromboembolism OR Thromboembolism, Pulmonary OR Thromboembolisms, Pulmonary) OR ("Venous Thrombosis"[Mesh] OR Phlebothrombosis OR Phlebothromboses OR Thrombosis, Venous OR Thromboses, Venous OR Venous Thromboses OR Deep Vein Thrombosis OR Deep Vein Thromboses OR Thromboses, Deep Vein OR Vein Thromboses, Deep OR Vein Thrombosis, Deep OR Deep-Venous Thrombosis OR Deep-Venous Thromboses OR Thromboses, Deep-Venous OR Thrombosis, Deep-Venous OR Deep-Vein Thrombosis OR Deep-Vein Thromboses OR Thromboses, Deep-Vein OR Thrombosis, Deep-Vein OR Thrombosis, Deep Vein OR Deep Venous Thrombosis OR Deep Venous Thromboses OR Thromboses, Deep Venous OR Thrombosis, Deep Venous OR Venous Thromboses, Deep OR Venous Thrombosis, Deep) OR (cardiovascular risk) OR (macrovascular diseases) OR ("Peripheral Vascular Diseases"[Mesh] OR Disease, Peripheral Vascular OR Peripheral Vascular Disease OR Vascular Disease, Peripheral OR Peripheral Angiopathies OR Angiopathies, Peripheral OR Angiopathy, Peripheral OR Peripheral Angiopathy OR Vascular Diseases, Peripheral OR Diseases, Peripheral Vascular) OR (cardiocerebrovascular disease) OR (cardiovascular death)) |
| **CINAHL** | TX ( (MH "Cardiovascular Diseases"OR Cardiovascular Disease OR Disease, Cardiovascular OR Diseases, Cardiovascular) OR (MH "Coronary Disease"OR Coronary Diseases OR Disease, Coronary OR Diseases, Coronary OR Coronary Heart Disease OR Coronary Heart Diseases OR Disease, Coronary Heart OR Diseases, Coronary Heart OR Heart Disease, Coronary OR Heart Diseases, Coronary OR coronary artery disease) OR (MH "Heart Diseases"OR Heart Disease OR Cardiac Diseases OR Cardiac Disease OR Cardiac Disorders OR Cardiac Disorder OR Heart Disorders OR Heart Disorder) OR (MH "Stroke"OR Strokes OR Cerebrovascular Accident OR Cerebrovascular Accidents OR CVA (Cerebrovascular Accident) OR CVAs (Cerebrovascular Accident) OR Cerebrovascular Apoplexy OR Apoplexy, Cerebrovascular OR Vascular Accident, Brain OR Brain Vascular Accident OR Brain Vascular Accidents OR Vascular Accidents, Brain OR Cerebrovascular Stroke OR Cerebrovascular Strokes OR Stroke, Cerebrovascular OR Strokes, Cerebrovascular OR Apoplexy OR Cerebral Stroke OR Cerebral Strokes OR Stroke, Cerebral OR Strokes, Cerebral OR Stroke, Acute OR Acute Stroke OR Acute Strokes OR Strokes, Acute OR Cerebrovascular Accident, Acute OR Acute Cerebrovascular Accident OR Acute Cerebrovascular Accidents OR Cerebrovascular Accidents, Acute) OR (MH "Cerebrovascular Disorders"OR Cerebrovascular Disorder OR Vascular Diseases, Intracranial OR Intracranial Vascular Disease OR Intracranial Vascular Diseases OR Vascular Disease, Intracranial OR Intracranial Vascular Disorders OR Intracranial Vascular Disorder OR Vascular Disorder, Intracranial OR Vascular Disorders, Intracranial OR Cerebrovascular Diseases OR Cerebrovascular Disease OR Disease, Cerebrovascular OR Diseases, Cerebrovascular OR Brain Vascular Disorders OR Brain Vascular Disorder OR Vascular Disorder, Brain OR Vascular Disorders, Brain OR Cerebrovascular Occlusion OR Cerebrovascular Occlusions OR Occlusion, Cerebrovascular OR Occlusions, Cerebrovascular OR Cerebrovascular Insufficiency OR Cerebrovascular Insufficiencies OR Insufficiencies, Cerebrovascular OR Insufficiency, Cerebrovascular) OR (MH "Atrial Fibrillation"OR Atrial Fibrillations OR Fibrillation, Atrial OR Fibrillations, Atrial OR Auricular Fibrillation OR Auricular Fibrillations OR Fibrillation, Auricular OR Fibrillations, Auricular OR Persistent Atrial Fibrillation OR Atrial Fibrillation, Persistent OR Atrial Fibrillations, Persistent OR Fibrillation, Persistent Atrial OR Fibrillations, Persistent Atrial OR Persistent Atrial Fibrillations OR Familial Atrial Fibrillation OR Atrial Fibrillation, Familial OR Atrial Fibrillations, Familial OR Familial Atrial Fibrillations OR Fibrillation, Familial Atrial OR Fibrillations, Familial Atrial OR Paroxysmal Atrial Fibrillation OR Atrial Fibrillation, Paroxysmal OR Atrial Fibrillations, Paroxysmal OR Fibrillation, Paroxysmal Atrial OR Fibrillations, Paroxysmal Atrial OR Paroxysmal Atrial Fibrillations) OR (MH "Rheumatic Heart Disease"OR Disease, Rheumatic Heart OR Diseases, Rheumatic Heart OR Heart Disease, Rheumatic OR Heart Diseases, Rheumatic OR Rheumatic Heart Diseases OR Bouillaud Disease OR Disease, Bouillaud OR Bouillaud's Disease OR Bouillauds Disease OR Disease, Bouillaud's) OR (MH "Myocardial Infarction"OR Infarction, Myocardial OR Infarctions, Myocardial OR Myocardial Infarctions OR Cardiovascular Stroke OR Cardiovascular Strokes OR Stroke, Cardiovascular OR Strokes, Cardiovascular OR Myocardial Infarct OR Infarct, Myocardial OR Infarcts, Myocardial OR Myocardial Infarcts OR Heart Attack OR Heart Attacks) OR (MH "Myocardial Ischemia"OR Ischemia, Myocardial OR Ischemias, Myocardial OR Myocardial Ischemias OR Ischemic Heart Disease OR Heart Disease, Ischemic OR Disease, Ischemic Heart OR Diseases, Ischemic Heart OR Heart Diseases, Ischemic OR Ischemic Heart Diseases) OR (MH "Heart Failure"OR Cardiac Failure OR Heart Decompensation OR Decompensation, Heart OR Heart Failure, Right-Sided OR Heart Failure, Right Sided OR Right-Sided Heart Failure OR Right Sided Heart Failure OR Myocardial Failure OR Congestive Heart Failure OR Heart Failure, Congestive OR Heart Failure, Left-Sided OR Heart Failure, Left Sided OR Left-Sided Heart Failure OR Left Sided Heart Failure) OR (MH "Pulmonary Heart Disease"OR Heart Disease, Pulmonary OR Heart Diseases, Pulmonary OR Disease, Pulmonary Heart OR Cor Pulmonale OR Diseases, Pulmonary Heart OR Pulmonary Heart Diseases) OR (MH "Pulmonary Embolism"OR Pulmonary Embolisms OR Embolism, Pulmonary OR Embolisms, Pulmonary OR Pulmonary Thromboembolisms OR Pulmonary Thromboembolism OR Thromboembolism, Pulmonary OR Thromboembolisms, Pulmonary) OR (MH "Venous Thrombosis"OR Phlebothrombosis OR Phlebothromboses OR Thrombosis, Venous OR Thromboses, Venous OR Venous Thromboses OR Deep Vein Thrombosis OR Deep Vein Thromboses OR Thromboses, Deep Vein OR Vein Thromboses, Deep OR Vein Thrombosis, Deep OR Deep-Venous Thrombosis OR Deep-Venous Thromboses OR Thromboses, Deep-Venous OR Thrombosis, Deep-Venous OR Deep-Vein Thrombosis OR Deep-Vein Thromboses OR Thromboses, Deep-Vein OR Thrombosis, Deep-Vein OR Thrombosis, Deep Vein OR Deep Venous Thrombosis OR Deep Venous Thromboses OR Thromboses, Deep Venous OR Thrombosis, Deep Venous OR Venous Thromboses, Deep OR Venous Thrombosis, Deep) OR (MH "Peripheral Vascular Diseases"OR Disease, Peripheral Vascular OR Peripheral Vascular Disease OR Vascular Disease, Peripheral OR Peripheral Angiopathies OR Angiopathies, Peripheral OR Angiopathy, Peripheral OR Peripheral Angiopathy OR Vascular Diseases, Peripheral OR Diseases, Peripheral Vascular) OR(cardiovascular event OR cardiovascular events) OR(cardiovascular risk) OR(macrovascular diseases) OR(cardiocerebrovascular disease) OR(cardiovascular death) ) **AND** TX ( (systemic immune inflammatory index OR systemic immune-inflammatory index or SII OR Systemic-immune-inflammation index OR Systemic immune-inflammation index OR neutrophil × platelets/ lymphocyte) ) |
| **Cochrane library** | #1 (systemic immune inflammatory index OR systemic immune-inflammatory index or SII OR Systemic-immune-inflammation index)  #2 MeSH descriptor: [Cardiovascular Diseases] explode all trees  #3 (Cardiovascular Diseases OR Cardiovascular Disease OR Disease, Cardiovascular OR Diseases, Cardiovascular)  #4 MeSH descriptor: [Coronary Disease] explode all trees  #5 (Coronary Disease OR Coronary Diseases OR Disease, Coronary OR Diseases, Coronary OR Coronary Heart Disease OR Coronary Heart Diseases OR Disease, Coronary Heart OR Diseases, Coronary Heart OR Heart Disease, Coronary OR Heart Diseases, Coronary OR coronary artery disease)  #6 MeSH descriptor: [Heart Diseases] explode all trees  #7 (Heart Diseases OR Heart Disease OR Cardiac Diseases OR Cardiac Disease OR Cardiac Disorders OR Cardiac Disorder OR Heart Disorders OR Heart Disorder)  #8 MeSH descriptor: [Stroke] explode all trees  #9 (Stroke OR Strokes OR Cerebrovascular Accident OR Cerebrovascular Accidents OR CVA (Cerebrovascular Accident) OR CVAs (Cerebrovascular Accident) OR Cerebrovascular Apoplexy OR Apoplexy, Cerebrovascular OR Vascular Accident, Brain OR Brain Vascular Accident OR Brain Vascular Accidents OR Vascular Accidents, Brain OR Cerebrovascular Stroke OR Cerebrovascular Strokes OR Stroke, Cerebrovascular OR Strokes, Cerebrovascular OR Apoplexy OR Cerebral Stroke OR Cerebral Strokes OR Stroke, Cerebral OR Strokes, Cerebral OR Stroke, Acute OR Acute Stroke OR Acute Strokes OR Strokes, Acute OR Cerebrovascular Accident, Acute OR Acute Cerebrovascular Accident OR Acute Cerebrovascular Accidents OR Cerebrovascular Accidents, Acute)  #10 MeSH descriptor: [Cerebrovascular Disorders] explode all trees  #11 (Cerebrovascular Disorders OR Cerebrovascular Disorder OR Vascular Diseases, Intracranial OR Intracranial Vascular Disease OR Intracranial Vascular Diseases OR Vascular Disease, Intracranial OR Intracranial Vascular Disorders OR Intracranial Vascular Disorder OR Vascular Disorder, Intracranial OR Vascular Disorders, Intracranial OR Cerebrovascular Diseases OR Cerebrovascular Disease OR Disease, Cerebrovascular OR Diseases, Cerebrovascular OR Brain Vascular Disorders OR Brain Vascular Disorder OR Vascular Disorder, Brain OR Vascular Disorders, Brain OR Cerebrovascular Occlusion OR Cerebrovascular Occlusions OR Occlusion, Cerebrovascular OR Occlusions, Cerebrovascular OR Cerebrovascular Insufficiency OR Cerebrovascular Insufficiencies OR Insufficiencies, Cerebrovascular OR Insufficiency, Cerebrovascular)  #12 MeSH descriptor: [Rheumatic Heart Disease] explode all trees  #13 (Rheumatic Heart Disease OR Disease, Rheumatic Heart OR Diseases, Rheumatic Heart OR Heart Disease, Rheumatic OR Heart Diseases, Rheumatic OR Rheumatic Heart Diseases OR Bouillaud Disease OR Disease, Bouillaud OR Bouillaud's Disease OR Bouillauds Disease OR Disease, Bouillaud's)  #14 MeSH descriptor: [Atrial Fibrillation] explode all trees  #15 (Atrial Fibrillation OR Atrial Fibrillations OR Fibrillation, Atrial OR Fibrillations, Atrial OR Auricular Fibrillation OR Auricular Fibrillations OR Fibrillation, Auricular OR Fibrillations, Auricular OR Persistent Atrial Fibrillation OR Atrial Fibrillation, Persistent OR Atrial Fibrillations, Persistent OR Fibrillation, Persistent Atrial OR Fibrillations, Persistent Atrial OR Persistent Atrial Fibrillations OR Familial Atrial Fibrillation OR Atrial Fibrillation, Familial OR Atrial Fibrillations, Familial OR Familial Atrial Fibrillations OR Fibrillation, Familial Atrial OR Fibrillations, Familial Atrial OR Paroxysmal Atrial Fibrillation OR Atrial Fibrillation, Paroxysmal OR Atrial Fibrillations, Paroxysmal OR Fibrillation, Paroxysmal Atrial OR Fibrillations, Paroxysmal Atrial OR Paroxysmal Atrial Fibrillations)  #16 MeSH descriptor: [Myocardial Infarction] explode all trees  #17 (Myocardial Infarction OR Infarction, Myocardial OR Infarctions, Myocardial OR Myocardial Infarctions OR Cardiovascular Stroke OR Cardiovascular Strokes OR Stroke, Cardiovascular OR Strokes, Cardiovascular OR Myocardial Infarct OR Infarct, Myocardial OR Infarcts, Myocardial OR Myocardial Infarcts OR Heart Attack OR Heart Attacks)  #18 (Myocardial Ischemia OR Ischemia, Myocardial OR Ischemias, Myocardial OR Myocardial Ischemias OR Ischemic Heart Disease OR Heart Disease, Ischemic OR Disease, Ischemic Heart OR Diseases, Ischemic Heart OR Heart Diseases, Ischemic OR Ischemic Heart Diseases)  #19 MeSH descriptor: [Myocardial Ischemia] explode all trees  #20 (cardiovascular event OR cardiovascular events)  #21 MeSH descriptor: [Heart Failure] explode all trees  #22 (Heart Failure OR Cardiac Failure OR Heart Decompensation OR Decompensation, Heart OR Heart Failure, Right-Sided OR Heart Failure, Right Sided OR Right-Sided Heart Failure OR Right Sided Heart Failure OR Myocardial Failure OR Congestive Heart Failure OR Heart Failure, Congestive OR Heart Failure, Left-Sided OR Heart Failure, Left Sided OR Left-Sided Heart Failure OR Left Sided Heart Failure)  #23 (Pulmonary Heart Disease OR Heart Disease, Pulmonary OR Heart Diseases, Pulmonary OR Disease, Pulmonary Heart OR Cor Pulmonale OR Diseases, Pulmonary Heart OR Pulmonary Heart Diseases)  #24 MeSH descriptor: [Pulmonary Heart Disease] explode all trees  #25 MeSH descriptor: [Pulmonary Embolism] explode all trees  #26 (Pulmonary Embolism OR Pulmonary Embolisms OR Embolism, Pulmonary OR Embolisms, Pulmonary OR Pulmonary Thromboembolisms OR Pulmonary Thromboembolism OR Thromboembolism, Pulmonary OR Thromboembolisms, Pulmonary)  #27 (Venous Thrombosis OR Phlebothrombosis OR Phlebothromboses OR Thrombosis, Venous OR Thromboses, Venous OR Venous Thromboses OR Deep Vein Thrombosis OR Deep Vein Thromboses OR Thromboses, Deep Vein OR Vein Thromboses, Deep OR Vein Thrombosis, Deep OR Deep-Venous Thrombosis OR Deep-Venous Thromboses OR Thromboses, Deep-Venous OR Thrombosis, Deep-Venous OR Deep-Vein Thrombosis OR Deep-Vein Thromboses OR Thromboses, Deep-Vein OR Thrombosis, Deep-Vein OR Thrombosis, Deep Vein OR Deep Venous Thrombosis OR Deep Venous Thromboses OR Thromboses, Deep Venous OR Thrombosis, Deep Venous OR Venous Thromboses, Deep OR Venous Thrombosis, Deep)  #28 MeSH descriptor: [Venous Thrombosis] explode all trees  #29 (cardiovascular risk)  #30 (macrovascular diseases)  #31 MeSH descriptor: [Peripheral Vascular Diseases] explode all trees  #32 (Peripheral Vascular Diseases OR Disease, Peripheral Vascular OR Peripheral Vascular Disease OR Vascular Disease, Peripheral OR Peripheral Angiopathies OR Angiopathies, Peripheral OR Angiopathy, Peripheral OR Peripheral Angiopathy OR Vascular Diseases, Peripheral OR Diseases, Peripheral Vascular)  #33 (cardiocerebrovascular disease)  #34 (cardiovascular death)  **#1 AND (#2 OR #3 OR #4 OR #5 OR #6 OR #7 OR #8 OR #9 OR #10 OR #11 OR #12 OR #13 OR #14 OR #15 OR #16 OR #17 OR #18 OR #19 OR #20 OR #21 OR #22 OR #23 OR #24 OR #25 OR #26 OR #27 OR #28 OR #29 OR #30 OR #31 OR #32 OR #33 OR #34)** |

| **TABLE S2 |** The quality assessment of included studies | | | | | | | | | |
| --- | --- | --- | --- | --- | --- | --- | --- | --- | --- |
| **Study (cohort)** | **Representativeness of exposed cohort** | **Selection of non-exposed cohort** | **Ascertainment of exposure** | **Outcome not present before study** | **Comparability** | **Assessment of outcome** | **Follow-up long enough*** | **Adequacy of follow up** | **Quality score** |
| Xu, 2021 | ★ | ★ | ★ | ★ | ★★ | ★ | ★ | ★ | 9 |
| Jin, 2021 | ★ | ★ | ★ | ★ | ★★ | ★ | ★ | ★ | 9 |
| Liu,2020 | ★ | ★ | ★ | ★ | ☆☆ | ★ | ☆ | ★ | 6 |
| Jiang, 2022 | ★ | ★ | ★ | ★ | ★★ | ★ | ☆ | ☆ | 7 |
| Zhang F, 2022 | ★ | ★ | ★ | ★ | ★★ | ★ | ☆ | ☆ | 7 |
| **Study (case-control)** | **Case definition** | **Representativeness of the cases** | **Selection of Controls** | **Definition of Controls** | **Comparability** | **Ascertainment of exposure** | **Same method of ascertainment** | **Non-Response rate** | **Quality score** |
| Zhang X, 2021 | ★ | ★ | ★ | ★ | ★★ | ★ | ★ | ★ | 9 |
| Tosu, 2021 | ★ | ★ | ★ | ★ | ★☆ | ★ | ★ | ★ | 8 |
| Liu, 2021 | ★ | ★ | ★ | ★ | ★★ | ★ | ★ | ★ | 9 |
| Karahan, 2021 | ★ | ★ | ★ | ★ | ☆☆ | ★ | ★ | ★ | 7 |
| Weng, 2021 | ★ | ★ | ★ | ★ | ★★ | ★ | ★ | ★ | 9 |
| Morga, 2020 | ★ | ☆ | ★ | ★ | ☆☆ | ★ | ★ | ★ | 6 |
| Zhang Z, 2022 | ★ | ★ | ★ | ★ | ★★ | ★ | ★ | ★ | 9 |
| Aydin, 2021 | ★ | ☆ | ★ | ★ | ☆☆ | ★ | ★ | ★ | 6 |
| * Median/mean follow-up of more than 3 years or maximum follow-up of more than 5 years was considered enough. | | | | | | | | | |

| **TABLE S3 |** Sensitivity analysis of the association between SII and CVD | | | | | |
| --- | --- | --- | --- | --- | --- |
| **Risk of CVD (high vs. low SII cohorts)** | | | | | |
| **Subgroup** | **No. of studies** | **HR** | **95% CI** | ***P*-value** | **Meta-regression (*P* _interaction_)** |
| **Region** |  |  |  |  |  |
| *China* | 7 | 1.38 | 1.18-1.61 | **<0.001** | 0.885 |
| *USA* | 1 | 1.51 | 1.18-1.93 | **0.001** |  |
| **Sample size** |  |  |  |  | 0.417 |
| *<5000* | 4 | 1.79 | 1.24-2.59 | **0.002** |  |
| *>5000* | 4 | 1.24 | 1.08-1.43 | **0.003** |  |
| **SII level differences (CVD cases vs. controls)** | | | | | |
| **Subgroup** | **No. of studies** | **WMD** | **95% CI** | ***P*-value** | **Meta-regression (*P* _interaction_)** |
| **Region** |  |  |  |  |  |
| *China* | 4 | 259.4 | 783.4-440.3 | **0.005** | 0.755 |
| *Turkey* | 3 | 589.3 | 168.3-1010.2 | **0.006** |  |
| *USA* | 1 | 103.8 | 36.3-171.3 | **0.003** |  |
| *Poland* | 1 | 793.9 | 635.6-952.2 | **<0.001** |  |
| **Sample size** |  |  |  |  | 0.436 |
| *<5000* | 8 | 390.5 | 261.3-519.8 | **<0.001** |  |
| *>5000* | 1 | 103.8 | 36.31-171.3 | **0.003** |  |
| SII, systemic immune-inflammation index; CVD, cardiovascular disease; HR, hazard ratio; WMD, weighted mean difference. | | | | | |
